# 3D-printed zeolite 13X-Strontium chloride units as ammonia carriers

**DOI:** 10.1016/j.heliyon.2023.e19376

**Published:** 2023-08-22

**Authors:** Nasir Shezad, Marco D'Agostini, Ali Ezzine, Giorgia Franchin, Paolo Colombo, Farid Akhtar

**Affiliations:** a Division of Materials Science, Department of Engineering Sciences and Mathematics, Luleå University of Technology, Luleå, SE-971 87, Sweden; b Department of Industrial Engineering, University of Padova, Via Marzolo, 9, Padova, 35131, Italy; c Department of Materials Science and Engineering, The Pennsylvania State University, University Park, PA, USA

**Keywords:** 3D printed structure, Ammonia, Zeolite, Alkaline earth metal halides, Physicochemical sorption, Kinetics

## Abstract

The selective catalytic reduction (SCR) system in automobiles using urea solution as a source of NH_3_ suffers from solid deposit problems in pipelines and poor efficiency during engine startup. Although direct use of high pressure NH_3_ is restricted due to safety concerns, which can be overcome by using solid sorbents as NH_3_ carrier. Strontium chloride (SrCl_2_) is considered the best sorbent due to its high sorption capacity; however, challenges are associated with the processing of stable engineering structures due to extraordinary volume expansion during the NH_3_ sorption. This study reports the fabrication of a novel structure consisting of a zeolite cage enclosing the SrCl_2_ pellet (SPZC) through extrusion-based 3D printing (Direct Ink Writing). The printed SPZC structure demonstrated steady sorption of NH_3_ for 10 consecutive cycles without significant uptake capacity and structural integrity loss. Furthermore, the structure exhibited improved sorption and desorption kinetics than pure SrCl_2_. The synergistic effect of zeolite as physisorbent and SrCl_2_ as chemisorbent in the novel composite structure enabled the low-pressure (<0.4 bar) and high-pressure (>0.4 bar) NH_3_ sorption, compared to pure SrCl_2,_ which absorbed NH_3_ at pressures above 0.4 bar. Regeneration of SPZC composite sorbent under evacuation showed that 87.5% percent of NH_3_ was desorbed at 20 °C. Thus, the results demonstrate that the rationally designed novel SPZC structure offers safe and efficient storage of NH_3_ in the SCR system and other applications.

## 1 Introduction

The combustion of fossil fuels releases various pollutants into the air, such as sulphur oxide (SOx), nitrogen oxide (NOx), and carbon oxide (COx). The mortalities during the COVID-19 pandemic have shown the link between pollution, especially NOx and particulate matter, and the coronavirus's mortality rate [1,2]. The major sources of NOx emission are fossil fuel power plants and the transport sector. A selective catalytic reduction system in the transport sector converts NOx into elemental N_2_ and water. Usually, this method uses urea solution to generate NH_3_, which acts as a reductant to reduce NOx into harmless compounds. The efficiency of the technique is quite good (around 95%), and it is successfully applied for commercial applications [3,4]. However, the urea solution cannot operate below −11 °C and freezes, which could damage the diesel exhaust fluid (DEF) filled tank. In addition, the process involves the hydrolysis of urea to produce NH_3_, which is slow during engine startup time due to lower temperatures in the SCR system. Thus, there is a delay in the supply of NH_3_ in the SCR reaction, which may let some NOx into the environment and may lead to deposit formation in the pipe lines [5]. The problem can be overcome by using solid NH_3_ carriers that supply NH_3_ without delay and can work at low temperatures without solid deposit formation [6,7]. These NH_3_ sorbent unit would be easy to replaced or recharged depending upon the type of sorbent material Also, the sorbent-based SCR system would be compact and lightweight compared to typical urea-based systems [8].

Due to their high capacity, alkaline earth metal halides (AEMHs) have been used for NH_3_ sorption. The sorption of NH_3_ over AEMHs proceeds through forming a metal ammine complex. Among AEMHs, SrCl_2_ has the highest NH_3_ uptake as it forms an amine complex with 8 molecules of NH_3_, for instance, compared to MgCl_2,_ which forms an amine complex with 6 molecules of NH_3_ [7]. Further, the binding energies of NH_3_ molecules with MgCl_2_ are higher than SrCl_2,_ and a high temperature is needed for regeneration [[8], [9], [10]]. During the ammonia sorption, AEMHs undergo expansion in volume, ca. 400% for MgCl_2_ and ca.300% for SrCl_2_ [11,12]. Therefore, most structured AEMHs adsorbents are unstable during ammonia sorption and disintegrate into powder. The powder formation can increase the pressure drop across the bed, which is detrimental to the adsorption system as it could damage the reaction chamber [13]. Additionally, the metal-amine complex formation occurs when NH_3_ gives its lone pair electrons to metal ions of AEMHs, thereby releasing exothermic heat of reaction which affects the thermal stability and, subsequently, the structural integrity of the sorbent. Thus, these challenges are the hurdle in fabricating stable and robust structures of the AEMHs.

Another challenge is the sluggish sorption kinetics of SrCl_2_ and low adsorption capacity at low pressure (below 0.4 bar) at normal conditions [7,8,12,14]. Recently, Cao et al. reported the structured SrCl_2_, SrCl_2_/graphite, SrCl_2_/rGO, and MOF composites using the freeze-casting method. Those composites showed good efficiency concerning NH_3_ uptake and sorption kinetics [12,14,15]. However, extraordinary volume changes are expected during NH_3_ sorption for typical structures and composites of AEMHs. Besides, freeze casting is a tedious and comparatively less efficient approach with the limited choice of solvent and to fabricate complex structures such as monoliths [16]. On the other hand, 3D printing is a modern approach to fabricating complex and highly efficient structures for various applications. The advantage of 3D printing technique is accuracy, smoothness of the structure, low waste material, and complex geometries [17]. Moreover, unlike freeze casting, in 3D printing, various types of complex structures can be designed and printed without special molds.

This study focuses on devising a concept of structured cages for SrCl_2_ to accommodate volume swings associated with NH_3_ sorption. These cages could provide the necessary free space for the volume expansion of the SrCl_2_ during the formation of the amine complex and act as a barrier for spreading powder. The capacity of SrCl_2_ remains intact, and it can absorb the same amount of NH_3_ as that of powder. Therefore, the disintegration of the SrCl_2_ unit inside the cage would not create structural or functional problems until it remained enclosed. Further, the selection of material for the cage is crucial. A good sorbent material like zeolite can be the best choice owing to its availability, stability, and ability to capture the NH_3_. Interestingly, zeolites adsorb NH_3_ at lower pressure, significantly below the working pressure of the SrCl_2_. Furthermore, zeolites capture the NH_3_ through physical adsorption rather than chemisorption [[18], [19], [20], [21]]. Therefore, combining zeolite and SrCl_2_ can improve the NH_3_ uptake and solve the problem of poor adsorption activity of SrCl_2_ at low-pressure conditions. In this work, we 3D printed a zeolite 13X cage enclosing SrCl_2_ pellet inside (SPZC) using a Direct Ink Writing technique. The sorbents were characterized using X-ray diffraction(XRD), nitrogen adsorption and desorption, scanning electron microscopy (SEM), and thermogravimetric analysis (TGA) technique. The sorbent demonstrated excellent NH_3_ uptake for 10 consecutive cycles without formation of loose particles or powder inside the sorption chamber. The kinetic data has shown that adding zeolite enhances the NH_3_ sorption at low-pressure regions.

## 2 Material and methods

### 2.1 Fabrication of pellets and zeolite cage

**Fig. 1:**
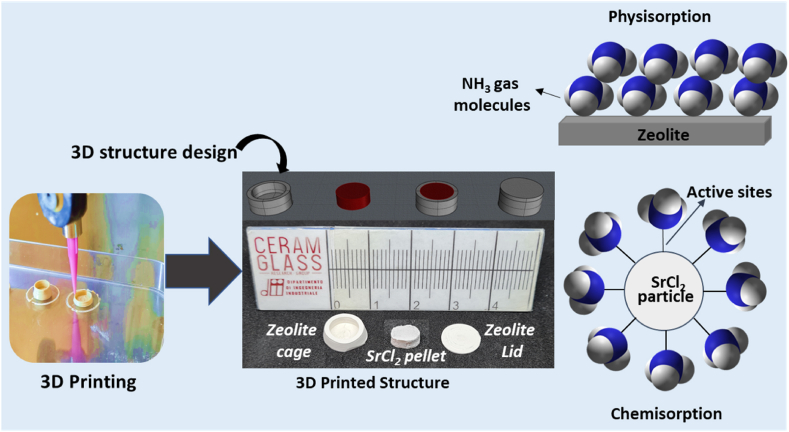
Photo of the 3D printing process, model design of the composite structure, photographs of the 3D printed zeolite cage and SrCl_2_ pellet, and a schematic of the sorption mechanisms on zeolite and SrCl_2_.

Zeolite 13X cages of cylindrical design (Ø10 × 5 mm) to contain the SrCl_2_ pellets during NH_3_ sorption were 3D printed by Direct Ink Writing. A schematic of the process is illustrated in Fig. 1. A potassium geopolymer with the molar composition of 3.8 SiO_2_ – Al_2_O_3_ – K_2_O synthesized from metakaolin (Argical 1200S, Imerys, France) and an alkaline solution of potassium silicate (Kasolv 205, PQ Corporation, The Netherlands), potassium hydroxide (Honeywell Research Chemicals, USA) and distilled water were chosen as the structural matrix for the zeolite particles. The alkaline solution was prepared in advance and aged for 12 h at 40 °C to allow for a complete dissolution of the reagents.

The slurry for Direct Ink Writing was prepared in an ice bath by adding the metakaolin and fillers to the alkaline solution under high-shear mechanical mixing. The temperature of the slurry was maintained close to 0 °C at all times to delay the onset of geopolymer polycondensation, which would have resulted in a rapid loss of workability. The composition of the solid fraction of the slurry, on a dry basis, is 60 wt% zeolite 13X (2–4 μm, Luoyang Jianlong Chemical Industry Co., Ltd), 3 wt% Na-bentonite (ClearOFF Minerals, UK) as a rheological additive, 2 wt% carboxymethylcellulose (Sigma, Germany) as a stabilizing agent and 35 wt% K-geopolymer; good printability was achieved with a solid-to-liquid ratio of 1.93 by weight. After 30 min of mixing, the slurry was transferred to a syringe (Vieweg, Germany) and defoamed on a high-energy planetary mixer (ARE-250, Thinky Corporation, Japan).

3D printing of the cages was carried out on a Direct Ink Writing printer (Delta WASP 2040 Turbo, WASP, Italy) equipped with an Auger screw extruder (LDM Extruder 3.0, WASP, Italy) and a 580 μm tapered nozzle (Vieweg, Germany). The syringe, refrigerated using a cooling jacket, acts as a reservoir from which the slurry is pushed into the extruder chamber by compressed air and finally deposited onto the build plate. After printing, the samples were cured for 7 days at room temperature and ≈100% R.H., then dried at 75 °C overnight and heat-treated at 450 °C for 02 h to remove the carboxymethylcellulose.

### 2.2 NH_3_ sorption experiments

The NH_3_ sorption measurements were performed on a High IsoSORP sorption apparatus (High-pressure TGA, TA instrument, New Castle, DE, USA). The device comprises a magnetic suspension balance, an electrical heater, a vacuum pump, and an oil bath. A certain amount of sample was loaded into the measuring chamber. The specimens were first degassed under evacuation at 150 °C for 03 h. After that, buoyancy measurement was performed using helium (purity 99.99%) to evaluate the mass and volume of the sample at 10 bar pressure and 20 °C. The system was purged 5 times before buoyancy measurement to ensure no residual adsorbate gas (NH_3_) from the previous experiments. Next, the gas was switched from He to NH_3_ with a setpoint pressure of 3 bar and 20 °C. Again, the system was purged 5 times before starting the experiment to ensure the gas pipeline has pure NH_3_ gas. Finally, the sorption experiment was carried out at 3 bar and desorption at 0 bar (vacuum). All the experiments were carried out under the same conditions, and 10 cycles were performed for the SrCl_2_ pellet inside the zeolite cage (SPZC). Thermal regeneration was done between the cycles, and the buoyancy test was measured separately.

### 2.3 Characterizations of sorbents

3D printed structures were characterised characterized using various analytical techniques. The microstructure of the material was investigated using a scanning electron microscope (Magellan 400 Extreme-High Resolution SEM, FEI Company, Eindhoven, Netherlands). Crystal structure and diffraction patterns were collected from the components ground into fine powders using CuKα radiation on an X-ray diffractometer (D8 Advance, Bruker Corporation, Germany) with a 0.02° step size and a 1.2°/min scan speed. The thermal stability of materials was analyzed using thermogravimetric analysis coupled with dynamic scanning calorimetry (TGA/DSC, NETSCH F690) up to 1000 °C using a heating rate of 10 K/min. Specific Surface Area was measured with an automated gas adsorption analyzer (Autosorb iQ, Anton Paar, Austria) using Ar at 87 K, as recommended by IUPAC guidelines for microporous materials [22]. Samples were degassed at 350 °C under a high vacuum for 08 h before analysis. The linear range for BET SSA calculation was selected at a relative pressure of 0.008–0.04 according to Rouquerol's correction for microporous materials.

## 3 Results and discussion

**Fig. 2:**
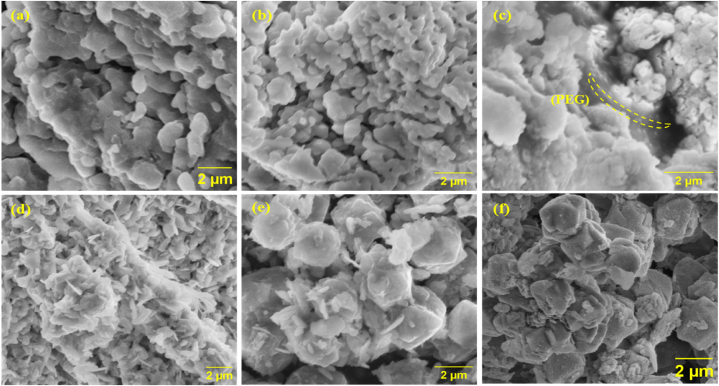
SEM micrographs of the sorbents; (a) SrCl_2_, (b) SrCl_2_ after NH_3_ sorption, (c) SrCl_2_ pellet. (d) SrCl_2_ pellet after NH_3_ sorption, (e) Zeolite cage (f) Zeolite cage after NH_3_ sorption.

Evaluating microstructural changes in the structured sorbent allows us to estimate the capacity and stability for NH_3_ sorption. The microstructure of SrCl_2_ salt and zeolite cage was analyzed before and after NH_3_ sorption measurements, as shown in Fig. 2. The SrCl_2_ showed the changes in microstructure. It has developed a porous structure after sorption measurement due to NH_3_ gas desorption. Other studies have also reported induced porosity leading to an increase in the surface area of SrCl_2_ [12]. In Fig. 2(c), the SrCl_2_ pellet confirms the presence of polyethylene glycol (PEG), interconnecting the SrCl_2_ particles. The SrCl_2_ pellet also develops a porous structure after NH_3_ adsorption and desorption, as shown in Fig. 2(d). After desorption, the SrCl_2_ pellet still demonstrates the interconnectivity of the salt particles owing to the presence of PEG as a binder. None of the images shows the melting spread problem of SrCl_2,_ which suggests that the structure is thermally stable during the NH_3_ sorption measurements. Fig. 2(e and f) displays the microstructure of the zeolite cage before and after NH_3_ sorption. The 13X crystals and geopolymer flakes can be seen without any change before and after cyclic sorption measurements.

**Fig. 3:**
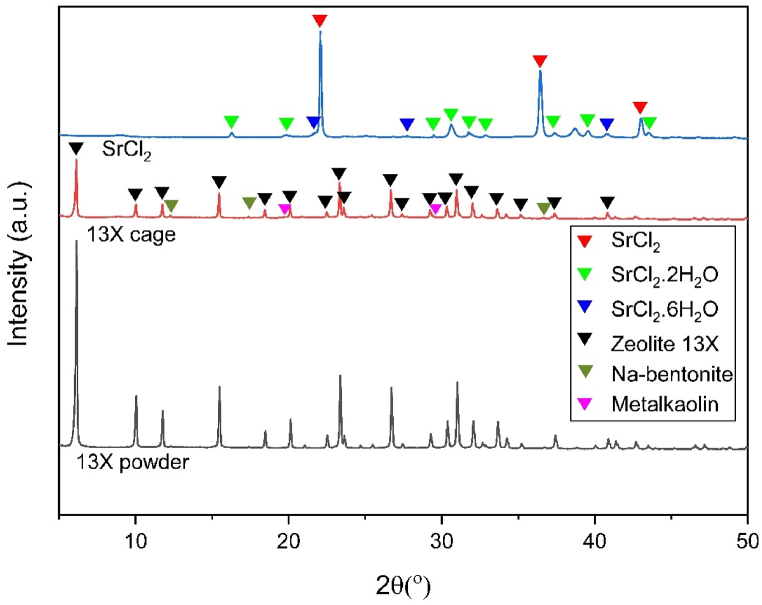
X-ray diffractograms of zeolite 13X powder, 3D printed 13X-geopolymer composite cage, and SrCl_2_ after activation at 150 °C.

The X-ray diffractogram of the zeolite-geopolymer cage (Fig. 3) displays all the reflections associated with the initial 13X powder, indicating excellent stability of the zeolite in the geopolymer slurry. Additional peaks were identified as Na-bentonite, which was employed as a rheological additive. Furthermore, the X-ray diffractogram highlights the presence of the geopolymer matrix in the form of a subtle amorphous halo centered around 27° together with a few diffractions associated with crystalline impurities (muscovite, anatase, and quartz) in the metakaolin, which do not take part in the geopolymerisation reaction. The SrCl_2_ powder was treated at 150 °C before NH_3_ sorption tests to simulate the activation process. Anhydrous SrCl_2_ was identified as the primary phase from the X-ray diffractograms (Fig. 2(b)), with the dihydrate and hexahydrate forms as minor constituents. A few unidentified peaks are most likely associated with the monohydrate form of SrCl_2_.

**Fig. 4:**
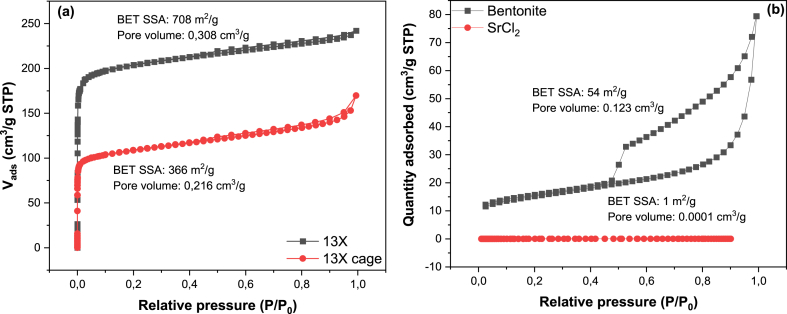
Argon adsorption-desorption isotherms for the zeolite 13X powder and 3D printed 13X-geopolymer composite cage measured at 87 K (a), and nitrogen adsorption-desorption isotherms for Na-bentonite and SrCl_2_ measured at 77 K (b).

Fig. 4 shows the adsorption isotherms to evaluate the surface area and pore structure. As shown in Fig. 4(a), both the initial 13X powder and the 13X-geopolymer composites display a Type I isotherm characterized by a high uptake of adsorbate at low pressure, indicating micropores. However, the composite also reveals the presence of some degree of macroporosity, as evidenced by the uptick of the isotherm as it approaches saturation. For the initial zeolite 13X powder, the SSA was 708 m^2^g^-1,^ and a pore volume of 0.308 cm^3^g^-1^ were measured, consistent with the data reported in the literature [23]. The values for the 13X-geopolymer composite cage were calculated as 366 m^2^g^-1^ and 0.216 cm^3^g^-1^ for SSA and pore volume, respectively. The SSA value is slightly lower than the theoretical value of the composite, assuming 60 wt% zeolites which are completely accessible to the adsorbate. The data may indicate a partial occlusion of the zeolites by the geopolymer matrix or by residual CMC, which was not entirely removed by heat treatment. The pore volume is higher than expected for the composite owing to the intrinsic macroporosity of the geopolymer matrix.

The isotherm for bentonite was measured using N_2_ at 77K, yielding a BET SSA value of 54 m^2^g^-1^ (P/P_0_ range of 0.03–0.15) and a pore volume of 0.123 cm^3^g^-1^. As shown in Fig. 4(b), the isotherm can be ascribed to a Type IV with a Type H3 hysteresis loop, indicating the presence of mesopores with a slit-shaped morphology. The shape of the isotherm at low P/P_0_ values suggests that a small fraction of large micropores may also be present [[24], [25], [26]]. No influence of bentonite on the textural properties of the composite could be detected, likely because of the small volume fraction used in the formulation. The SrCl_2_ powder, measured with N_2_ at 77 K, yielded an SSA of 1 m^2^g^-1^, indicating the essentially non-porous nature of the material [12].

**Fig. 5:**
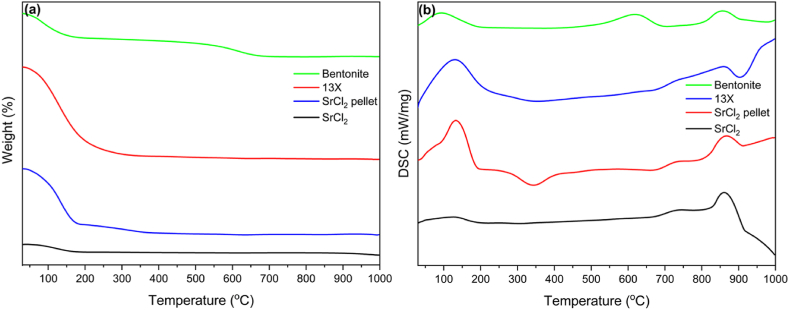
(a) Thermogravimetric (TGA) plots (b) Differential scanning calorimetry (DSC) plots of bentonite, zeolite 13X, SrCl_2_ powder, and pellet.

The thermal stability of the crystal structures of zeolite and SrCl_2_ was evaluated through TGA-DSC analysis, as shown in Fig. 5 (a and b). All of the samples displayed good stability. The SrCl_2_ showed two small peaks, visible in the DSC plot below 200 °C corresponding to stepwise dehydration and removal of free moisture [27,28]. There is a peak at 875 °C which shows the melting of SrCl_2_. The melting spread has been reported as a problem in the NH_3_ sorption process owing to the high exothermic heat of the amine complex formation. The SrCl_2_ pellet shows more significant peaks around 130 °C, which could be the melting of PEG-2000 and the dehydration of SrCl_2._ The 13X showed moisture removal below 200 °C and an exothermic peak at 907 °C possibly due to phase transformation [29]. At high temperatures, typically above 800 C, the crystalline 13X zeolite transforms into an amorphous aluminosilicate, and the amorphous phase recrystallizes into a nepheline phase by releasing the energy with the temperature increase [[30], [31], [32]]. The bentonite showed two peaks apart from free moisture loss below 200 °C. The peak at 632 °C represents the loss of lattice water, and a second peak at 857 °C corresponds to the presence of a small quantity of montmorillonite and illite in bentonite clay [33]. As per TGA/DSC results, all the components of SPZC structure are stable till 800 °C, therefore, thermal treatment of the structure (at 450 °C as mentioned in methodology section) would not affect the crystallinity of the materials.

**Fig. 6:**
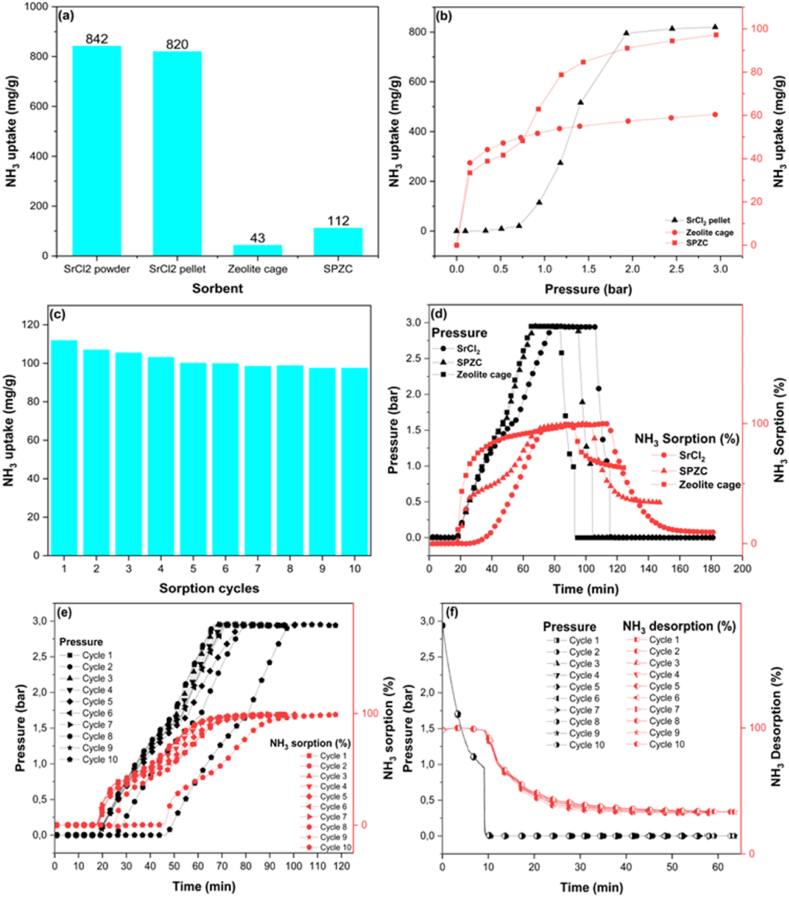
(a) The NH_3_ uptake capacity of SrCl_2_ powder, SrCl_2_ pellet, zeolite cage, and SPZC structure at 20 °C and 3 bar. (b) Effect of pressure on NH_3_ sorption capacity of the SrCl_2_ pellet, zeolite cage, and SPZC structure. (c) Cyclic NH_3_ sorption measurements for SPZC structure. (d) NH_3_ sorption kinetics plots of adsorption, desorption, and pressure for SrCl_2_ powder, zeolite cage, and SPZC structure at 20 °C. (e) The pressure curves and NH_3_ sorption % of SPZC structure, for 10 cyles of NH_3_ sorption from vacuum to 3 bar at 20 °C. (f) The pressure curves and NH_3_ sorption % of SPZC structure for 10 cylcles of NH_3_ desorption from 3 bar to vacuum at 20 °C.

Fig. 6(a) shows the screening experiments showing the performance of various components of the designed structure. The sourced SrCl_2_ powder exhibited the highest NH_3_ sorption capacity of 842 mg/g. The structured SrCl_2_ pellet showed the maximum NH_3_ uptake of 820 mg/g, corresponding to the mass composition of SrCl_2_ in the pellet. The SrCl_2_ involves adsorption of the NH_3_ through the chemisorption process, which proceeds through the formation of a metal-amine complex as shown in equation (1):

(1) $${SrCl}_{2}+{8NH}_{3}↔Sr{\left(NH_{3}\right)}_{8}Cl_{2}$$

where 8 molecules of NH_3_ are bonded to the SrCl_2_. The empty zeolite cage showed an NH_3_ uptake of 43 mg/g, which is low compared to the SrCl_2_. The zeolites capture the NH_3_ employing van der Waals force (dipole moment of NH_3_ molecules) and electrostatic attraction of exchangeable cations that attach the gas molecules with adsorbent particles [32]. For the SPZC, the sorption capacity is higher than zeolite but lower than the pellet. The sorption capacity is calculated by measuring the amount of solute (NH_3_) adsorb per gram of the adsorbent. In SPZC, the weight of the pellet is low compared to the zeolite cage. Therefore, a high NH_3_ capacity of the pellet is shadowed by calculating the capacity per gram. In the SPZC structure concept, a calculated free space exists inside the cage, so the overall structure remains intact during sorption. The SrCl_2_ expands three times during the adsorption of NH_3_, so the SPZC structure can accommodate the volume changes without losing the overall structural integrity and capacity. In addition, an exothermic metal-amine formation reaction generates heat which on accumulation can cause sintering or melting of the sorbent. In the SPZC composite structure, the 3D printed zeolite cage was found stable before and after ammonia sorption, and it showed the characteristic crystals of 13X zeolite, Fig. 2 e, and f, suggesting the structural stability and potential of utilization of SPZC for various purposes.

Pressure is an important parameter in a gas adsorption system. Fig. 6(b) shows the effect of pressure on the NH_3_ uptake of different adsorbents. Equilibrium pressure (p) for SrCl_2_ at reaction temperature can be estimated using Van't Hoff equation as shown below:

(2) $$\ln\left(p\right)=-\frac{\Delta H}{RT}+\frac{\Delta S}{R}$$

where T is the temperature, R is the universal gas constant, ΔH is the enthalpy of adsorption, and ΔS is the entropy. Reported values of ΔH and ΔS are 41.4 kJ mol^−1^ and 228.8 J mol^−1^ K^−1^, respectively [34]. Using equation (2), value of equilibrium pressure for SrCl_2_ at 20 °C is 0.4 bar. The SrCl_2_ pellet doesn't show any NH_3_ uptake below 0.4 bar pressure, which could be a drawback for low-pressure applications. The capacity of adsorbents increases with pressure, and for the SrCl_2_ pellet, sorption increases exponentially at higher pressure values. Equation (1) shows the decrease in moles from reactants to the product. Therefore, an increase in pressure will lead to the coordination of more NH_3_ molecules to cations of metal halides and a higher rate of metal-amine complex formation. For the zeolite cage, adsorption dominates at the low-pressure region, and 65% of NH_3_ adsorption occurs below 0.2 bar. It increases with an increase in pressure, but the effect of the rise in pressure is not so significant after 0.6 bar. The highly microporous adsorbents are characterized by physisorption in at low pressure due to van der Waals forces and electrostatic attraction [18,35].The SPZC structure shows adsorption at low and high pressure [36] owing to the synergistic effect of the sorption properties of zeolites and AEMHs. The curve at low pressure is analogous to that of the zeolite curve [[37], [38], [39]]. Thus, the SPZC demonstrated the potential to be used for NH_3_ sorption for applications from low and high-pressure.

Fig. 6(c) shows the cyclic adsorption capacity of the SPZC structure for 10 consecutive cycles. During the cyclic measurements, the sample was regenerated, followed by buoyancy measurements and NH_3_ sorption under the same conditions. However, buoyancy was not needed for the cycles when the chamber was kept closed. As shown in Table 1, there is almost no change in the sorption capacity of the structure. Nevertheless, a little decrease can be seen between the first few cycles, which could be due to melting and recrystallization of the PEG binder during cyclic measurement leading to surface coverage of the SrCl_2_ particles and absorbed NH_3_ on zeolites which cannot be recovered only with the application of evacuation conditions. A slight decrease (6.3%) in NH_3_ sorption of SrCl_2_ has also been reported by Brynjarsson et al. due to salt's repeated thermal expansion (1300 cycles), which created separate particles from the bed in the thermal energy storage system [40]. However, Cao et al. have reported an unchanged sorption capacity of SrCl_2_ composites for cyclic experiments [12]. The cyclic experiments show that the SPZC structure can be regenerated and used to >90% of the sorption capacity for industrial applications without needing replacement.

**Table 1:** Cyclic performance of SPZC structure for NH_3_ sorption and desorption.

Sorption cycles	NH_3_ uptake (mg/g)	Sorption time (min)	Desorption time (min)
1	112	77	52
2	107	71	52
3	106	71	52
4	103	71	57
5	100	82	58
6	100	77	57
7	98	71	58
8	99	71	57
9	98	71	63
10	98	71	64

In the SCR system, NH_3_ is injected into the engine exhaust gases, and then the mixture passes over a catalyst where NOx emissions are transformed into N_2_ and H_2_O. The continuous supply of the reductant is essential, therefore, the rate of NH_3_ charging (adsorption) and discharging (desorption) cycle is very important. Therefore, faster sorption kinetics are highly desired characteristics of sorbent. The kinetic sorption data showing an increase in pressure and percent NH_3_ adsorption for different materials is plotted in Fig. 6(d and e) for different materials and cyclic adsorption data for SPZC structure. For the SrCl_2_ powder, the sample showed no uptake of NH_3_ below 0.4 bar which is the equilibrium pressure for SrCl_2_ powder at 20 °C calculated from Van't Hoff equation. Meanwhile, SrCl_2_ exhibited an increased uptake with pressure and reached saturation in 81 min. It shows the sluggish kinetics of NH_3_ adsorption of AEMHs. The zeolite cage shows a steep increase in capacity at lower pressure below 0.4 bar, which continues to increase until the material reaches saturation capacity. The steep curve shows the adsorption of NH_3,_ where molecules quickly find the sites over a high surface area microporous zeolite 13X sorbent [6,41]. The zeolite13X reached 80% of the saturation capacity in 35 min, whereas SrCl_2_ reached the same saturation level in 65 min owing to fast sorption kinetics for the physisorption process. In the SPZC structure, both components contribute differently according to the sorption mechanisms. The SPZC took 61 min to reach 80% of the saturation capacity, less than powder samples and longer than zeolite. The SPZC structure exhibited two different sorption mechanisms; (1) physisorption and (2) chemisorption. The sharp increase in NH_3_ uptake at the low-pressure region is due to physical adsorption by zeolite. On the other hand, the NH_3_ uptake continues to slowly increase at high-pressure regions owing to dominant chemisorption by SrCl_2_. The synergistic effect of physical and chemical sorption contributed to the improved kinetics, an additional advantage of adopting the current approach. Besides, volume expansion is completely accommodated in the logical design of SPZC structure.

The adsorption mechanism of NH_3_ molecules involves the adsorption on the surface, overcoming the surface mass transfer resistance and diffusing into bulk sites immediately below the surface and then further diffusion into the bulk of material [39]. Therefore, the diffusivity of each sorbent was estimated using the following equation (3):

(3) $$1-\theta \approx \frac{6}{\pi^{2}}\exp\left(-\frac{\pi^{2}Dt}{r^{2}}\right)$$

where θ is the fractional adsorption (ratio of adsorption of solute at any time t to the maximum adsorption at equilibrium), r is the radius of the adsorbent's crystal, and D is the diffusivity. The diffusivity for SrCl_2_, SPZC, and zeolite cage was estimated using the reported method [12,35]. The calculated values of diffusion time constant are 9.15 × 10^−5^, 6.79 × 10^−4,^ and 5.99 × 10^−4^ s^−1^ for SrCl_2_, SPZC, and zeolite cage, respectively. The data shows higher mass transfer kinetics of SPZC structure compared to SrCl_2_ powder. The zeolite having a porous structure showed the highest diffusion time constant value, thereby quickly reaching saturation capacity. However, the drawback of this approach is the limitation of data fitting in specific pressure regions. The mass transfer coefficient of SrCl_2_ was estimated for an adsorption capacity of <5%, whereas zeolite model fitting was performed for adsorption greater than 70% [12,34,42].

Desorption kinetics in Fig. 6(d, f) shows desorption took place with a decrease in pressure without applying heat for different materials and cyclic data for SPZC structure. The NH_3_ molecules from the SrCl_2_.8NH_3_ complex were desorbed to SrCl_2_.NH_3_, by removing the pressure to vacuum conditions, which shows the salt's practicality for easy regeneration. The sample can be reused without undergoing any changes in structure. In addition, monoamine SrCl_2_.NH_3_ has been reported as a stable phase at STP and needs thermal treatment for regeneration back to SrCl_2_ [43]. The NH_3_ molecules attached to the SrCl_2_ has different bond length, the monoamine has shorter bond length than octa-amine; therefore, a high amount of energy is needed to remove the monoamine from SrCl_2_ [39]. The time required to regenerate SrCl_2_ powder was 76 min. Meanwhile, the zeolite cage and SPZC structure were regenerated in 42 and 54 min, respectively. The desorption time for SrCl_2_ was longer than the zeolite cage and SPZC structure which may be due to sluggish kinetics owing to chemisorbed NH_3_ molecules. Considering the working capacity without thermal treatment, SrCl_2_ can readsorb 7 molecules of NH_3,_ approximately 87% of the saturation capacity at given pressure conditions. Therefore, thermal regeneration can be completely avoided, and time for regeneration could be decreased, which is necessary for commercial applications. Vacuum regeneration has also been reported by Liu et al. where it was observed that evacuation cannot fully regenerate the sorbent [38]. Similarly, Kubota et al. reported that desorption for the metal ammine complex depends on the temperature and concentration of NH_3_ [44]. This study demonstrated that removing NH_3_ concentration, i.e., applying a vacuum, could be a more efficient way to regenerate the adsorbent. Overall, the rational design of SPZC structures demonstrates the potential for commercial application in the SCR system and H_2_ carrier in the form of NH_3_.

## 4 Conclusion

In summary, we have shown that a rationally designed 3D-printed novel SPZC structure can effectively overcome the volume expansion challenge in the NH_3_ storage over AEMHs. The novel structure showed stable sorption performance for numerous cycles without changing the microstructure, as analyzed by the SEM. The synergistic effect of physicochemical adsorption owing to the SrCl_2_ and zeolite enabled the better performance of the SPZC structure at different pressure with improved sorption and mass transfer kinetics. Further, applying evacuation conditions avoided the thermal energy needed for regeneration. The sorbent recovered and reached 87.5% of the working capacity after vacuum regeneration, rendering the process economical and efficient. Hence, rationally designed AEMHs-based NH_3_ sorbents unit can potentially replace the urea solution in the SCR system and ensure the smooth injection of NH_3_ into SCR reaction chamber at different ambient conditions leading to efficient NOx reduction.

## Author contribution statement

Nasir Shezad; Marco D'Agostini; Ali Ezzine: Performed the experiments; Analyzed and interpreted the data; Wrote the paper. Giorgia Franchin; Paolo Colombo; Farid Akhtar: Conceived and designed the experiments; Analyzed and interpreted the data; Contributed reagents, materials, analysis tools or data; Wrote the paper.

## Data availability statement

Data will be made available on request.

## Declaration of competing interest

The authors declare that they have no known competing financial interests or personal relationships that could have appeared to influence the work reported in this paper.
